# Anti-Obesity Activities of the Compounds from *Perilla frutescens* var. *acuta* and Chemical Profiling of the Extract

**DOI:** 10.3390/molecules29112465

**Published:** 2024-05-23

**Authors:** Isoo Youn, Donglan Piao, Jisu Park, Seung A Ock, Sujin Han, Ah-Reum Han, Sunhye Shin, Eun Kyoung Seo

**Affiliations:** 1 Graduate School of Pharmaceutical Sciences, College of Pharmacy, Ewha Womans University, Seoul 03760, Republic of Korea; iyoun@ewha.ac.kr (I.Y.); parkdl@ewhain.net (D.P.); sujinh94@gmail.com (S.H.); 2 Advanced Radiation Technology Institute, Korea Atomic Energy Research Institute, Jeongeup 56212, Republic of Korea; jspark94@cnu.ac.kr (J.P.); arhan@kaeri.re.kr (A.-R.H.); 3 Department of Food and Nutrition, Seoul Women’s University, Seoul 01797, Republic of Korea; oktmddk@swu.ac.kr

**Keywords:** *Perilla frutescens* var. *acuta*, flavonoid diglucuronide, rosmarinic acid, adipogenesis, thermogenesis, UPLC-MS/MS, feature-based molecular networking, Progenesis QI

## Abstract

*Perilla frutescens* var. *acuta* (Lamiaceae) is widely used not only as an oil or a spice, but also as a traditional medicine to treat colds, coughs, fever, and indigestion. As an ongoing effort, luteolin-7-*O*-diglucuronide (**1**), apigenin-7-*O*-diglucuronide (**2**), and rosmarinic acid (**3**) isolated from *P. frutescens* var. *acuta* were investigated for their anti-adipogenic and thermogenic activities in 3T3-L1 cells. Compound **1** exhibited a strong inhibition against adipocyte differentiation by suppressing the expression of *Pparg* and *Cebpa* over 52.0% and 45.0%, respectively. Moreover, **2** inhibited the expression of those genes in a dose-dependent manner [*Pparg*: 41.7% (5 µM), 62.0% (10 µM), and 81.6% (50 µM); *Cebpa*: 13.8% (5 µM), 18.4% (10 µM), and 37.2% (50 µM)]. On the other hand, the *P. frutescens* var. *acuta* water extract showed moderate thermogenic activities. Compounds **1** and **3** also induced thermogenesis in a dose-dependent manner by stimulating the mRNA expressions of *Ucp1*, *Pgc1a*, and *Prdm16*. Moreover, an LC-MS/MS chromatogram of the extract was acquired using UHPLC-MS^2^ and it was analyzed by feature-based molecular networking (FBMN) and the Progenesis QI software (version 3.0). The chemical profiling of the extract demonstrated that flavonoids and their glycoside derivatives, including those isolated earlier as well as rosmarinic acid, are present in *P. frutescens* var. *acuta*.

## 1. Introduction

Obesity has arisen as a worldwide concern because it significantly increases the risk of diseases, including type 2 diabetes mellitus (T2-DM), dyslipidemia, hypertension, dementia, and cancers [1]. Anti-obesity medications (AOMs), such as orlistat and phentermine, have long been used to treat patients, and recently, glucagon-like peptide 1 (GLP1) agonists have been introduced in the market for obesity treatment [2]. Although these medications exhibit a potent efficacy to treat obesity, they still cause several side effects, such as cardiovascular and gastrointestinal symptoms. Therefore, efforts to find different AOM targets are necessary as patients with obesity need long-term treatments.

Testing anti-obesity effects can be divided into two approaches: anti-adipogenesis and adipose thermogenesis. Anti-adipogenic activity refers to the inhibition against the differentiation of preadipocytes into mature adipocytes and lipid accumulation [3]. This process is mainly mediated by two transcription factors, peroxisome proliferator-activated receptor γ (PPAR-γ) and CCAAT/enhancer-binding protein α (C/EBP-α). On the other hand, adipose thermogenesis helps to increase energy expenditure by facilitating heat generation from the stored energy, and the transcription factors related to this process include PPAR-γ coactivator 1α (PGC-1α), PR domain-containing 16 (PRDM16), and uncoupling protein 1 (UCP1) [4]. 

The dried leaves of *Perilla frutescens* (L.) var. *acuta* (Thunb.) Kudô (“Perilla herb”, “Ja So Yeop” in Korea, Lamiaceae) is used to treat colds, coughs, fever, and indigestion in Asian traditional medicine [5]. It has been reported that it shows anti-inflammatory, antibacterial, antifungal, antidepressant, and cytotoxic activities [6]. In phytochemical studies, it has been known to have terpenoids, anthocyanins, flavonoids, phenylpropanoids, and fatty acids [7]. Thomas et al. showed the anti-adipogenic activity of a *P. frutescens* var. *acuta* extract in 3T3-L1 preadipocytes using a high-fat diet mouse model [8], and Choi et al. demonstrated that the *P. frutescens* var. *acuta* extract improves the pathology of metabolic syndrome and non-alcoholic fatty acid liver disease in a mouse model [9]. However, finding biologically active entities in this plant has not been widely studied to develop novel AOMs.

We have recently reported the anti-inflammatory activities of luteolin-7-*O*-diglucuronide (**1**), apigenin-7-*O*-diglucuronide (**2**), and rosmarinic acid (**3**) isolated from *P. frutescens* var. *acuta* in LPS-treated RAW 264.7 cells through the NF-κB signaling pathway [10]. As a follow-up to our previous study, a *P. frutescens* var. *acuta* water extract (PFW) and **1**–**3** were tested for their anti-adipogenic and thermogenic activities in 3T3-L1 cells, the most commonly used cell line for adipocyte research [11], in the current study. The anti-obesity activities were evaluated by measuring the gene expressions of *Pparg*, *Cebpa*, *Ucp1*, *Pgc1a*, and *Prdm16* compared to that of *18s*. We also aimed to systematically investigate the constituents of the PFW extract using UHPLC-MS/MS fragmentation and molecular networking (MN) approaches.

## 2. Results

### 2.1. Inhibitory Activity against Adipocyte Differentiation

**Figure 1:**
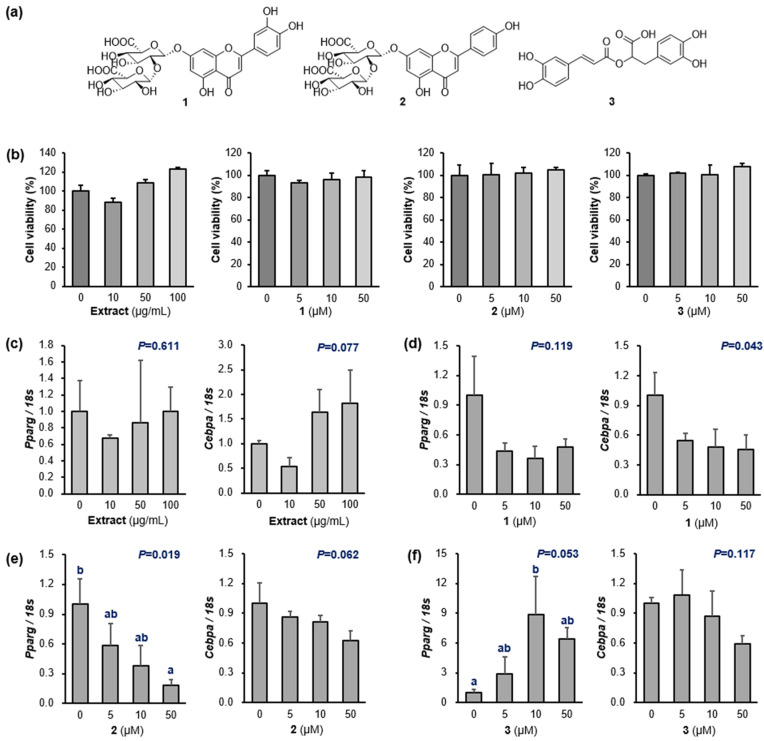
(**a**) Structures of luteolin-7-*O*-diglucuronide (**1**), apigenin-7-*O*-diglucuronide (**2**), and rosmarinic acid (**3**). (**b**) Cell viability of the *P. frutescens* var. *acuta* water extract (PFW) and **1**–**3** against a 3T3-L1 cell line. (**c**) Inhibitory effect against the preadipocyte differentiation of PFW (0, 10, 50, and 100 μg/mL). (**d**–**f**) Inhibition of preadipocyte differentiation by **1**–**3** (0, 5, 10, and 50 μM). 3T3-L1 preadipocytes were treated with each component dissolved in DMSO during differentiation. The mRNA levels were determined by quantitative real-time polymerase chain reaction (RT-PCR) with normalization relative to *18s* rRNA. Data are presented as means ± standard error of mean (*n* = 3). Different letters (a and b) indicate significant differences at *p* < 0.05 by Duncan’s multiple comparison test. *Pparg*, peroxisome proliferator-activated receptor gamma; *Cebpa*, CCAAT/enhancer-binding protein α; *P*, *p*-value.

The isolation and structural identification of **1**–**3** (Figure 1a) were reported in our previous study [10]. The effects of the PFW extract and **1**–**3** on the viability of 3T3-L1 preadipocytes were measured, and all of them did not exhibit cell cytotoxicity (>88.6% of the cells were alive and functioning) (Figure 1b and Table S1). Then, the expression of genes related to adipocyte differentiation was evaluated using real-time qPCR measurements. Although the PFW extract did not show a clear reduction in *Pparg* and *Cebpa* (Figure 1c), compound **1** strongly downregulated the expression of both *Pparg* [56.6% (5 μM); 63.8% (10 μM); and 51.7% (50 μM)] and *Cebpa* [45.0% (5 μM); 51.9% (10 μM); and 54.6% (50 μM)] (Figure 1d). Compounds **2** also suppressed the expression of *Pparg* and *Cebpa* in a dose-dependent manner (Figure 1e). However, the mRNA expressions of *Pparg* and *Cebpa* were not decreased by **3** (Figure 1f). The percentages of the anti-adipogenic effects are shown in Table S2. 

### 2.2. Thermogenesis of the Extract and ***1**–**3***

**Figure 2:**
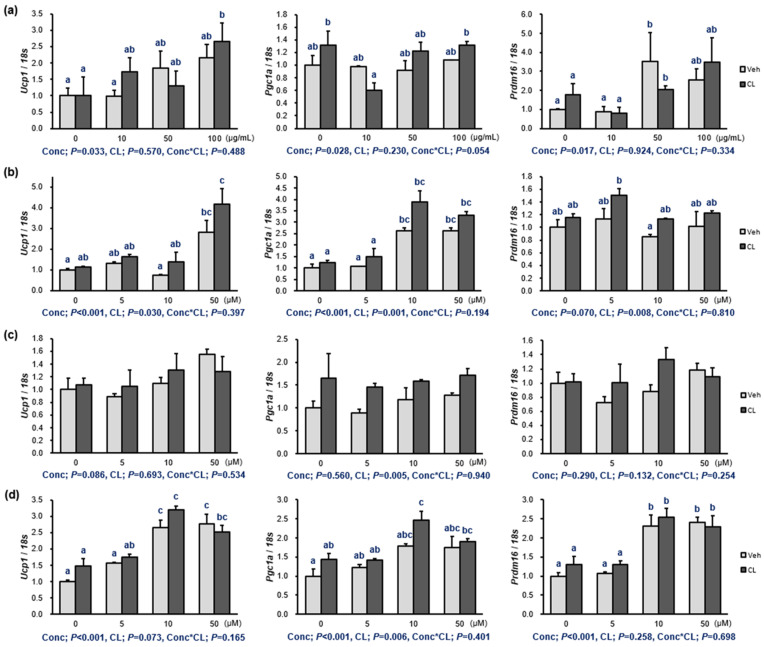
Thermogenetic induction of (**a**) the PFW extract (0, 10, 50, and 100 μg/mL) and (**b**–**d**) **1**–**3** (0, 5, 10, and 50 μM) on 3T3-L1 adipocytes. The compounds were identified as luteolin-7-*O*-diglucuronide (**1**), apigenin-7-*O*-diglucuronide (**2**), and rosmarinic acid (**3**). Fully differentiated 3T3-L1 preadipocytes were treated with each component dissolved in DMSO for 24 h with a 4 h treatment of CL316,243 (CL; 10 μM). The mRNA levels were determined by quantitative RT-PCR with normalization relative to 18s rRNA. Data are presented as means ± standard error of mean (*n* = 3). A two-way ANOVA was conducted to determine the effects of the concentration of the PFW extract and **1**–**3** (Conc), CL treatment (CL), and the interaction of Conc and CL (Conc*CL). Different letters (a, b and c) indicate significant differences at *p* < 0.05 by Duncan’s multiple comparison test. *Ucp1*, uncoupling protein 1; *Pgc1a*, peroxisome proliferator-activated receptor gamma coactivator 1-α; *Prdm16*, PR domain-containing 16; *P*, *p*-value.

Activating adipose thermogenesis exerts beneficial effects on the body fat content and metabolic health; therefore, the induction of thermogenesis by drugs can be a start to investigate its potential as an obesity treatment. To evaluate thermogenesis induction by the PFW extract and **1**–**3,** fully differentiated 3T3-L1 adipocytes were treated with the PFW extract and **1**–**3** in addition to CL316,243 (CL). CL is a β_3_-adrenoreceptor agonist that induces adipose thermogenesis, and we tried to determine the additional induction of thermogenesis by the PFW extract and **1**–**3** (Figure 2 and Table S3). The extract itself upregulated *Ucp1*, *Pgc1a*, and *Prdm16* in a dose-dependent manner. Compounds **1** and **2** showed a modest induction on the expression of *Ucp1* and *Pgc1a*. Three concentrations (5, 10, and 50 μM) of **3** showed a dose–response relationship in the stimulation of the gene expressions (*Ucp1*, *Pgc1a*, and *Prdm16*). In general, CL stimulated the action of the components on the thermogenic gene expressions.

### 2.3. Feature-Based Molecular Networking of the Extract

**Figure 3:**
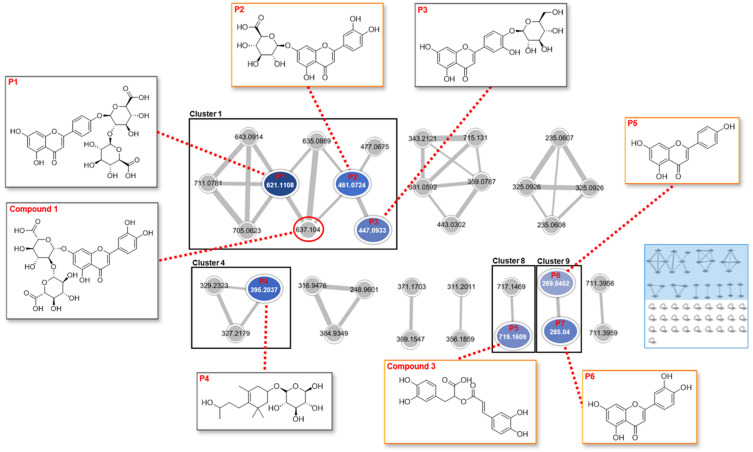
Feature-based molecular networking (FBMN) of the PFW extract. A total of 34 single nodes and 10 clusters (nodes ≥ 2) were found in the network, and a total of seven compounds (**3** and **P1**–**P6**) were identified using the FBMN analysis. Compounds in the orange boxes (**3**, **P2**, **P5**, and **P6**) indicate the components that were simultaneously identified by the FBMN and Progenesis QI (ProQI) analyses. Although the node “637.104” in **Cluster 1** was not identified in the FBMN analysis, it was assigned to compound **1** (luteolin-7-*O*-diglucuronide/luteolin-7-*O*-[*β*-D-glucuronosyl-(1→2)-*β*-D-glucuronide]) by the ProQI software ver. 3.0.

As demonstrated in Figure 3 and Table 1, 315 mass ions extracted from the Progensis QI (ProQI) software (version 3.0) were organized into a feature-based molecular networking (FBMN), which included 34 single nodes and 10 clusters (nodes ≥2). A total of seven compounds (**3** and **P1–P6**) from the PFW extract were characterized based on the global natural products social molecular networking (GNPS) library, and **Clusters 1**, **4**, **8**, and **9** corresponded to flavonoid glycosides, phenylpropanoid glycosides, polyphenols, and flavonoids, respectively. Although **P5** (apigenin) and **P6** (luteolin) were connected to each other in **Cluster 9**, they did not show correlations with the nodes in **Cluster 1**, which represents a flavonoid-related cluster. More details can be found on the GNPS website https://gnps.ucsd.edu/ProteoSAFe/status.jsp?task=105dc5b8472b4fe39b8b2fccbf4ca55b (accessed on 10 October 2023). The compounds in the orange boxes (**3**, **P2**, **P5**, and **P6**) were found simultaneously using the FBMN and ProQI approaches.

**Figure 4:**
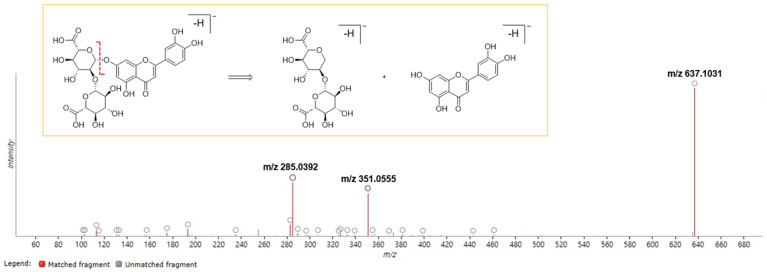
Example of the component prediction of the extract using ProQI. Compound **1** was identified as luteolin-7-*O*-diglucuronide by analyzing the fragmentation patterns of the peak “*m*/*z* 637.1031”. This peak was divided into two substructures: a flavonoid (*m*/*z* 285.0392) and a diglucuronide (*m*/*z* 351.0555).

The identification of potential chemical markers in the PFW extract was also carried out with the retention behavior and mass assignment of the components using ChemSpider and Element Composition in the ProQI software ver. 3.0. The candidate compounds were selected on the basis of matching score (≥40), fragmentation score (30), mass error (<5 ppm), and isotope similarity (≥90), and finally five chemical markers (**1**, **3**, **P2**, **P5**, and **P6**) were identified (Table S4). Although the node “637.104” of **Cluster 1** in Figure 3 was connected with the other flavonoid nodes, the FBMN analysis did not identify any known compound to this node. Later, it was identified as compound **1** (luteolin-7-*O*-diglucuronide/luteolin-7-*O*-[*β*-D-glucuronosyl-(1→2)-*β*-D-glucuronide]) with the assistance of ProQI by considering the fragmented ions “*m*/*z* 351.0555” and “*m*/*z* 285.0392” at the retention time of 6.07 min (Figure 4).

## 3. Discussion and Conclusions

Phenolic compounds, widely used as food colorants, have received attention since researchers have shown the relationship of phenolic-rich food intake and the decrease in the incidence of several diseases, such as cardiovascular diseases and cancers [12]. Moreover, several clinical studies have been conducted for the anti-obesity activity of phenolic compounds [13]. For example, epigallocatechin gallate (EGCG) in green tea and anthocyanins in *Hibiscus sabdariffa* have shown positive effects on the reduction in body weight, body mass index (BMI), and body fat.

To the best of our knowledge, this study reports for the first time that **1** and **2** suppressed adipocyte differentiation by reducing the mRNA expressions of *Pparg* and *Cebpa*. The differentiation of preadipocytes into mature adipocytes plays a key role in the development of obesity [14]. PPAR-γ has been known to be indispensable in the differentiation of adipocytes [15,16], and C/EBP-α is also very important in the late stage of adipocyte differentiation. The target genes of PPAR-γ and C/EBP-α are upregulated in this process, and lipids are accumulated in adipocytes [17]. As **1** and **2** did not show activity as PPAR-γ agonists in the previous study [10], we investigated the effects of **1** and **2** on the mRNA expression level of *Pparg*. Although **3** (50, 100, and 200 μM) suppressed the protein activities of PPAR-γ and C/EBP-α [18], it did not downregulate the mRNA expressions of those transcription factors at concentrations of 5, 10, and 50 μM in this study. While Thomas et al. reported that the extract of *P. frutescens* var. *acuta* suppressed the mRNA expressions of *Pparg* and *Cebpa* at concentrations of 0.1, 0.2, and 0.4 mg/mL [8], the inhibition of those mRNAs was not observed at concentrations of 10, 50, and 100 μg/mL in this study. 

Although *P. frutescens* (L.) Britton has been reported to promote thermogenesis through the p38 mitogen-activated protein kinase (MAPK) and phosphatidylinositol 3-kinase (PI3K)/Akt pathways, the biologically active components in the extract have never been investigated. The compounds **1** and **3** showed strong thermogenic induction by upregulating the mRNA expressions of *Ucp1*, *Pgc1a*, and *Prdm16*, while **2** mildly stimulated the thermogenesis of 3T3-L1 cells. The thermogenic activities of **1**–**3** are reported for the first time in this study. 

In addition to the isolation of pure compounds (**1**–**3**) from the PFW extract, the chemical profiling of the extract was performed in this study. Tandem mass (MS/MS) spectrometry-based molecular networking (MN) is a tool to identify ingredients in extracts or mixtures. In FBMN, the feature detection and alignment of spectra are applied to the acquired LC-MS/MS data, and then the results are produced as a feature quantification table and MS^2^ spectral summary [19]. These results are uploaded into the GNPS web platform for MN and compound identification. Compared to the classical GNPS-MN, FBMN discriminates isomers with similar MS/MS data, which are distinguished by the retention time or ion mobility separation. In this study, the FBMN analysis of the PFW extract resulted in the identification of seven compounds, which were mainly apigenin and luteolin derivatives. Although the FBMN results did not assign the accurate structure to each node in the network, the analysis helped to improve spectral annotation and identify the clusters of specific compound types in the plant material. The same LC-MS^2^ data were analyzed again using ChemSpider and Elemental Composition in ProQI according to the accurate mass, isotope distribution, and fragmentation pattern. The compounds **3**, **P2**, **P5**, and **P6** were simultaneously identified by the FBMN and ProQI approaches. However, **2** was not found in either analysis. 

Several studies confirmed the correlation of obesity with inflammation [20]. In an obese status, macrophages accumulated in the white adipose tissue release pro-inflammatory cytokines, including interleukins IL-1β, IL-6, IL-8, and IL-29, and tumor necrosis factor α (TNF-α), thus causing obesity-related inflammation [21]. Obesity also changes adipokine secretion. The plasma level of leptin with pro-inflammatory properties tends to increase, and that of adiponectin with anti-inflammatory functions decreases in obese individuals [22,23]. In our previous study, we demonstrated that **1**–**3** have anti-inflammatory activities via the suppression of NF-κB activity and the downregulation of the mRNA expressions of NF-κB target genes, including *Il6*, *Mcp1*, and *Tnfa* [10]. In this paper, we also found that compounds **1**–**3** isolated from *P. frutescens* var. *acuta* have anti-obesity effects, indicating that **1**–**3** would be good candidates for drug development for obesity treatments. However, the underlying mechanisms for their anti-obesity activities need to be elucidated in further studies. 

## 4. Materials and Methods

### 4.1. Plant Materials and Sample Preparation

The dried leaves of *P. frutescens* var. *acuta* were provided by Megabiosoop in Seoul, Republic of Korea, in April 2019 and a voucher specimen (No. EA387) was stored in the Natural Product Chemistry Laboratory, College of Pharmacy, Ewha Womans University, Seoul, Republic of Korea.

The plant material (2 kg) was extracted with water (20 L) for 15 h at room temperature, and then the solvent was evaporated in vacuo at 40 °C to obtain a concentrated water extract (352.78 g). Then, 5 mg of the PFW extract was transferred to a 1.5 mL vial and dissolved in 1 mL of 70% of LC-MS-grade methanol.

### 4.2. Cell Culture

The 3T3-L1 preadipocytes (Korea Cell Line Bank, Seoul, Republic of Korea) were grown in DMEM containing 4500 mg/L glucose and L-glutamine (Sigma-Aldrich, St. Louis, MO, USA) supplemented with 10% calf serum (Gibco, Waltham, MA, USA), 1% penicillin–streptomycin (Sigma-Aldrich) at 37 °C, and 5% CO_2_. Two days after confluence, cell differentiation was induced with DMEM with 10% fetal bovine serum (FBS; Sigma-Aldrich), 1.7 μM insulin (Sigma-Aldrich), 1 μM dexamethasone (Sigma-Aldrich), and 500 μM 3-isobutyl-1-methylxanthine (IBMX; Sigma-Aldrich). After 2 days, the medium was changed to DMEM with 10% FBS and 1.7 μM insulin, and after another 2 days, it was changed to DMEM with 10% FBS. 

### 4.3. Cell Viability

The cell viability of the 3T3-L1 cells was determined by 3-(4,5-dimethylthiazolyl-2)-2,5-diphenyl tetrazolium bromide (MTT; Alfa Aesar, Haverhill, MA, USA). At 70% confluence, 3T3-L1 preadipocytes were treated with the PFW extract (0, 10, 50, and 100 μg/mL) or **1**–**3** (0, 5, 10, and 50 μM) for 24 h. After aspirating the cell culture medium, the cells were incubated in DMEM with 10% FBS and 5 mg/mL MTT solution. After 1 h of incubation, the concentration of formazan, a purple product converted from a tetrazolium salt by the viable cells, was measured using a spectrophotometer at 595 nm.

### 4.4. Anti-Adipogenic and Thermogenic Gene Expression

To determine the anti-adipogenic effects of the PFW extract and **1**–**3**, the 3T3-L1 cells were treated with the PFW extract (0, 10, 50, and 100 μg/mL) or **1**–**3** (0, 5, 10, and 50 μM) during differentiation. To determine the thermogenic effects of the PFW extract and **1**–**3**, the fully differentiated 3T3-L1 adipocytes were treated with the PFW extract (0, 10, 50, and 100 μg/mL) or **1**–**3** (0, 5, 10, and 50 μM) for 24 h with a 4 h treatment of CL316,243 (CL; 10 μM; Sigma-Aldrich). After the treatment, the total RNA was extracted from the cells using the Trizol reagent (Invitrogen, Waltham, CA, USA), and cDNA was synthesized from 1 μg of total RNA using the PrimeScript II 1st strand cDNA synthesis kit (Takara Bio, Shiga, Japan). The mRNA levels of *Pparg*, *Cebpa*, *Ucp1*, *Pgc1a*, and *Prdm16* were quantified using TB Green Premix Ex Taq (Takara) and a StepOnePlus Real-time PCR System (Applied Biosystems, Waltham, MA, USA), and then normalized relative to 18S rRNA. The fold changes in gene expression were calculated by the ΔΔCt method. The specific primer sequences used are shown in Table S5.

### 4.5. Statistical Analysis

A one-way or two-way analysis of variance (ANOVA) with Duncan’s post hoc test was performed to determine significant differences among groups. To determine the anti-adipogenic effects of the PFW extract and **1**–**3**, a one-way ANOVA was conducted. To determine the thermogenic effects, a two-way ANOVA was conducted for the effects of the PFW extract or **1**–**3**, CL, and the interaction. The data are presented as the means ± SEM and were analyzed using the SPSS software (Version 26, IBM, Armonk, NY, USA).

### 4.6. UPLC-MS/MS Analysis

#### 4.6.1. UHPLC and MS/MS Conditions

LC-MS/MS data acquisition was conducted using a Waters ACQUITY UPLC system (Waters Corporation, Milford, MA, USA) connected to a SYNAPT XS quadrupole time-of-flight (Q-TOF) mass spectrometer (Waters Corporation). The separation of the components was performed on a BEH C18 chromatography column (2.1 × 100 mm, 1.7 μm, Waters Corporation). Mobile phase was (A) water with 0.1% formic acid and (B) acetonitrile with 0.1% formic acid, and the gradient elution was as follows: 0–2.5 min, 5% B; 2.5–21 min, 5–100% B; 21–26 min, 100% B; and 26.1–30 min, 5% B. The column oven was set to 40 °C and the autosampler temperature to 15 °C. The flow rate was 0.4 mL/min and injection volume was 1 μL. Comprehensive mass spectra information was acquired using MS^E^ in the negative mode. The parameters of the MS^E^ mode were set as follows: mass range, 100–1200 Da; capillary voltage, 2.2 kV; sampling cone voltage, 50 eV; source offset voltage, 30 eV; source temperature, 120 °C; desolvation temperature, 450 °C; cone gas flow rate, 50 L/h; and desolvation gas flow rate, 800 L/h. Nitrogen and argon were applied as cone and collision gases, respectively. The collision energy was 20–40 eV for high-energy function, and the scan time was 0.5 s. The data were calibrated in real time using a leucine enkephalin solution (*m*/*z* 554.2615 [M-H]^−^) as an external reference (LockSpray™) at a flow rate of 5 μL/min. The MassLynx v4.1 software (Waters Corporation) was used for data acquisition. The total chromatogram of the PFW extract is shown in Figure S1. 

#### 4.6.2. Feature-Based Molecular Networking

The MS^E^ data acquired in the negative mode were imported into the ProQI software (version 3.0) and processed for feature detection and alignment. FBMN was constructed following the online FBMN-ProQI workflow (https://ccms-ucsd.github.io/GNPSDocumentation/featurebasedmolecularnetworking-with-progenesisQI/, accessed on 10 October 2023) [19]. Both the feature quantification table (CSV file) and MS/MS spectral summary (MSP file) of the processed MS^E^ data were exported and uploaded into the GNPS platform (https://gnps.ucsd.edu, accessed on 10 October 2023) using WinSCP (https://winscp.net, accessed on 10 October 2023). The parameters for molecular networking were as follows: precursor ion mass tolerance and fragment ion mass tolerance, 0.02 Da; minimum cosine score, 0.70; minimum matched fragment ions, 6; maximum number of neighbor nodes for one single node, 10; and maximum size of a spectral family, 100. Cytoscape ver. 3.9.1 was used for network visualization and network analysis.

#### 4.6.3. Identification of Chemical Markers

LC-MS/MS data were imported into the ProQI software v3.0 (Waters Corporation) and processed for alignment. The selected adduct ions were M-H_2_O-H, M-H, 2M-H, M+K-2H, and M+FA-H in the negative mode. The parameters for peak picking were as follows: intensity > 20,000; spectral width > 0.3 min. The identification of compounds was performed using the ChemSpider and Elemental Composition search engines equipped in ProQI according to the accurate mass, isotope distribution, and fragmentation pattern. 

## Figures and Tables

**Table 1:** Compounds identified by the FBMN analysis.

	t*_R_* (min)	*m*/*z*	Adduct	Cosine Score	Mass Error (ppm)	Compound Name
**3**	7.68	719.1609	2M-H	0.86	1.53	Rosmarinic acid
**P1**	6.55	621.1108	M-H	0.76	1.28	Apigenin-4′-*O*-[(*β*-D-glucuronosyl-(1→2)-*β*-D-glucuronide]
**P2**	6.92	461.0724	M-H	0.85	1.32	Luteolin-7-*O*-D-glucuronide
**P3**	6.46	447.0933	M-H	0.87	0.68	Luteolin-4′-*O*-glucoside
**P4**	9.94	395.2037	M+Na-2H	0.71	9.34	Tsangane L-3-glucoside
**P5**	9.39	269.0452	M-H	0.84	1.81	Apigenin
**P6**	8.61	285.0400	M-H	0.88	0.00	Luteolin

## Data Availability

Data are contained within the article and Supplementary Materials.

## Supplementary Materials

The following supporting information can be downloaded at: https://www.mdpi.com/article/10.3390/molecules29112465/s1. Table S1: Cell viability of the *Perilla frutescens* var. *acuta* water extract (PFW) and **1**–**3**; Table S2: Anti-adipogenic effects of the PFW extract and **1**–**3** on 3T3-L1 cells; Table S3: Thermogenic effects of the PFW extract and **1**–**3** on 3T3-L1 cells; Table S4: Compounds identified using the Progenesis QI software (ver. 3.0); Table S5: Primer sequences for the in vitro assays; Figure S1: UPLC-MS/MS chromatogram of the PFW extract.
