# Supplementary material for: Anti-Obesity Activities of the Compounds from Perilla frutescens var. acuta and Chemical Profiling of the Extract
Source: Molecules. 2024 May 23;29(11):2465. doi: 10.3390/molecules29112465 (PMC11174005; doi:10.3390/molecules29112465)
Supplement: Supplementary file 1 [file molecules-29-02465-s001.zip › molecules-3013733-supplementary.pdf]

Anti-obesity activities of the compounds from *Perilla frutescens* var. *acuta* and chemical profiling of the extract

Isoo Youn[1,†], Donglan Piao[1,†], Jisu Park[2], Seung A Ock[3], Sujin Han[1], Ah-Reum Han[2], Sunhye Shin[3,*], and Eun Kyoung Seo[1,*]

[1]Graduate School of Pharmaceutical Sciences, College of Pharmacy, Ewha Womans University, Seoul, Korea
[2]Advanced Radiation Technology Institute, Korea Atomic Energy Research Institute, Jeongeup, Korea
[3]Department of Food and Nutrition, Seoul Women's University, Seoul, Korea
[†]The authors equally contributed to this work.

Table of contents

Table S1. Cell viability of the *Perilla frutescens* var. *acuta* water extract (PFW) and **1-3**……….………….……………..…..2

Table S2. Anti-adipogenic effects of PFW and **1-3** on 3T3-L1 cells……………....……………..…………….……….………..2

Table S3. Thermogenic effects of PFW extract and **1-3** on 3T3-L1 cells………...………………….……………...………….3

Table S4. Identified compounds from the Progenesis QI software……………….……….……………………….....…3

Table S5. Primer sequences for the in vitro assays……………………………..……………..…..……………….…3

Figure S1. UPLC-MS/MS chromatogram of PFW……………………………………….……….....…………………4

Table S1. Cell viability of PFW and **1**-**3**.

Conc. (μg/mL)	PFW (%)	Conc. (μM)	**1** (%)	**2** (%)	**3** (%)
0	100.0	0	100.0	100.0	100.0
10	88.6	5	93.6	100.5	101.9
50	108.6	10	96.4	102.4	100.4
100	123.2	50	98.3	105.2	108.0

Table S2. Anti-adipogenic effects of PFW and **1**-**3** on 3T3-L1 cells.

Conc. (μg/mL)	PFW (%)		Conc. (μM)	**1** (%)		**2** (%)		**3** (%)	
	Pparg/*18s*	*Cebpa*/*18s*		*Pparg*/*18s*	*Cebpa*/*18s*	*Pparg*/*18s*	*Cebpa*/*18s*	*Pparg*/*18s*	*Cebpa*/*18s*
10	32.4	46.2	5	56.6	45.0	41.7	13.8	-190.2	-8.7
50	-18.7	-63.1	10	63.8	51.9	62.0	18.4	-783.5	13.1
100	-13.9	-82.4	50	51.7	54.6	81.6	37.2	-544.6	41.0

Table S3. Thermogenic effects of PFW and **1-3** on 3T3-L1 cells.

Conc. (μg/mL)		PFW			Conc. (μM)		1			2			3		
		Ucp1/18s	*Pgc1a/18s*	*Prdm16/18s*			*Ucp1/18s*	*Pgc1a/18s*	*Prdm16/18s*	*Ucp1/18s*	*Pgc1a/18s*	*Prdm16/18s*	*Ucp1/18s*	*Pgc1a/18s*	*Prdm16/18s*
10	Veh	0.99	0.98	0.88	5	Veh	1.31	1.06	1.14	0.89	0.88	0.73	1.56	1.22	1.07
	CL	1.73	0.46	0.45		CL	1.43	1.21	1.31	0.97	0.88	1.00	1.19	1.00	1.00
50	Veh	1.84	0.92	3.52	10	Veh	0.73	2.61	0.86	1.09	1.18	0.88	2.66	1.79	2.31
	CL	1.30	0.93	1.15		CL	1.21	3.14	0.99	1.22	0.96	1.31	2.16	1.73	1.95
100	Veh	2.16	1.08	2.57	50	Veh	2.82	2.63	1.01	1.55	1.28	1.18	2.76	1.74	2.41
	CL	2.62	1.00	1.95		CL	3.65	2.67	1.06	1.19	1.03	1.07	1.71	1.33	1.76

Table S4. Identified compounds from the Progenesis QI software.

No.	t_R (min)	Adduct ion	m/z	Fragment information	Compound name	Formula	Score	Fragmentation score	Mass error (ppm)	Isotope similarity
**1**	6.07	M-H	637.1031	637.1031, 351.0555, 285.0392	Luteolin-7-*O*-[*β*-D-glucuronosyl-(1→2)-*β*-D-glucuronide]	$C_{27}H_{26}O_{18}$	48.5	52.0	-1.00	91.95
**3**	7.68	2M-H	719.1622	719.1622, 359.0789, 161.0249	Rosmarinic acid	$C_9H_8O_4$	41	27.5	0.46	94.76
**P2**	6.92	M-H	461.0725	461.0725, 285.0393	Luteolin-7-*O*-glucuronide	$C_{21}H_{18}O_{12}$	43.7	30.9	-0.27	90.61
**P5**	9.39	M-H	269.0450	269.0450, 117.0340	Apigenin	$C_{15}H_{10}O_5$	43.0	20.5	-1.17	96.09
**P6**	8.61	M-H	285.0400	285.0400, 151.0033, 133.0288	Luteolin	$C_{15}H_{10}O_6$	48.4	48.0	-1.60	96.02

Table S5. Primer sequences for the in vitro assays.

	Forward Primer	Reverse Primer
18S	ATC CCT GAG AAG TTC CAG CA	CCT CTT GGT GAG GTC GAT GT
Pparg	TTG ACC CAG AGC ATG GTG C	GAA GTT GGT GGG CCA GAA TG
Cebpa	AAT GGC AGT GTG CAC GTC TA	CCC CAG CCG TTA GTG AAG AG
Ucp1	GGG CCC TTG TAA ACA ACA AA	GTC GGT CCT TCC TTG GTG TA
Pgc1a	GTC CTT CCT CCA TGC CTG AC	GTG TGG TTT GCT GCA TGG TT
Prdm16	GGC TCA AGG AGG AGG AGA GA	AGG TCC GGG TCA GGT TCA TA

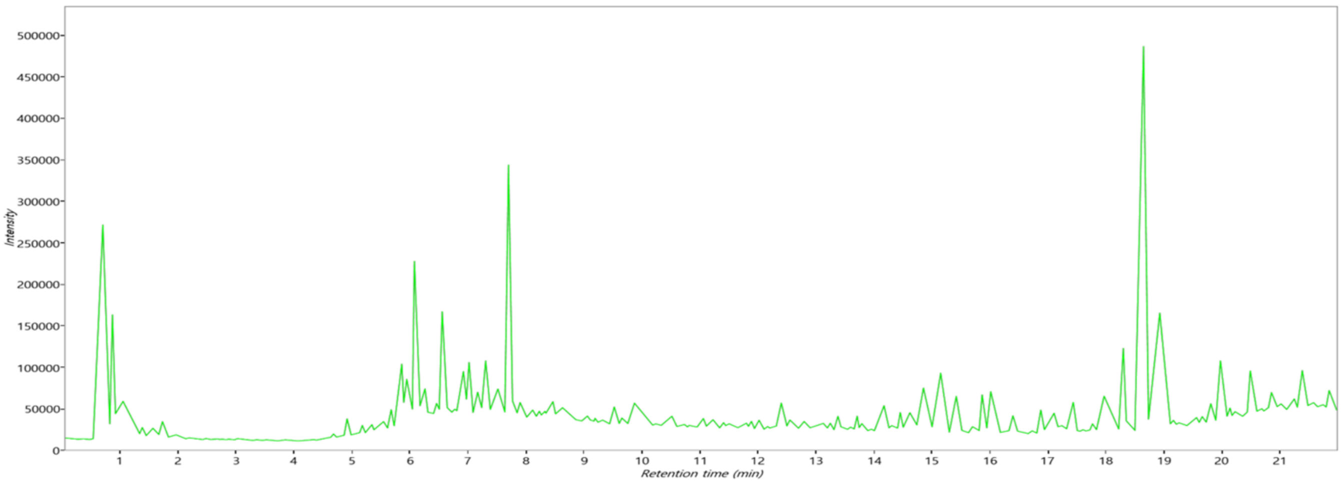

Figure S1. UPLC-MS/MS chromatogram of PFW.
